# Integrative analyses of a mitophagy-related gene signature for predicting prognosis in patients with uveal melanoma

**DOI:** 10.3389/fgene.2022.1050341

**Published:** 2022-12-05

**Authors:** Yanhua Cheng, Jingying Liu, Huimin Fan, Kangcheng Liu, Hua Zou, Zhipeng You

**Affiliations:** Jiangxi Province Division of National Clinical Research Center for Ocular Diseases, Jiangxi Clinical Research Center for Ophthalmic Disease, Jiangxi Research Institute of Ophthalmology and Visual Science, Affiliated Eye Hospital of Nanchang University, Nanchang, Jiangxi, China

**Keywords:** uveal melanoma, mitophagy, prognostic biomarker, immune infiltration, TCGA

## Abstract

We aimed to create a mitophagy-related risk model *via* data mining of gene expression profiles to predict prognosis in uveal melanoma (UM) and develop a novel method for improving the prediction of clinical outcomes. Together with clinical information, RNA-seq and microarray data were gathered from the Cancer Genome Atlas (TCGA) and Gene Expression Omnibus (GEO) databases. ConsensusClusterPlus was used to detect mitophagy-related subgroups. The genes involved with mitophagy, and the UM prognosis were discovered using univariate Cox regression analysis. In an outside population, a mitophagy risk sign was constructed and verified using least absolute shrinkage and selection operator (LASSO) regression. Data from both survival studies and receiver operating characteristic (ROC) curve analyses were used to evaluate model performance, a bootstrap method was used test the model. Functional enrichment and immune infiltration were examined. A risk model was developed using six mitophagy-related genes (*ATG12, CSNK2B, MTERF3, TOMM5, TOMM40, and TOMM70*), and patients with UM were divided into low- and high-risk subgroups. Patients in the high-risk group had a lower chance of living longer than those in the low-risk group (*p* < 0.001). The ROC test indicated the accuracy of the signature. Moreover, prognostic nomograms and calibration plots, which included mitophagy signals, were produced with high predictive performance, and the risk model was strongly associated with the control of immune infiltration. Furthermore, functional enrichment analysis demonstrated that several mitophagy subtypes may be implicated in cancer, mitochondrial metabolism, and immunological control signaling pathways. The mitophagy-related risk model we developed may be used to anticipate the clinical outcomes of UM and highlight the involvement of mitophagy-related genes as prospective therapeutic options in UM. Furthermore, our study emphasizes the essential role of mitophagy in UM.

## Introduction

Uveal melanoma (UM) is the most prevalent primary intraocular malignancy in adults, affecting 5–10 per million people (Ortega et al., 2020). The disease is confined to the eye in over 95% of patients at initial diagnosis; however, up to 50% of patients eventually develop metastasis, which is associated with poor prognosis and a survival of less than one year (Nathan et al., 2015; Castet et al., 2019). The bleak prognosis of UM has seen minimal improvement despite intensive research into its physiopathology, histology, and molecular biology (Ortega et al., 2020). Therefore, UM management currently focuses on the early identification of diagnostic and prognostic biomarkers and therapeutic targets.

Autophagy is a catabolic process that plays a vital role in maintaining homeostasis in various biological processes (Chung et al., 2020). Mitophagy, a specific type of autophagy, is a cellular process that removes aged and damaged mitochondria through lysosomal degradation (Ma et al., 2020). Mitophagy defects are linked to the onset and progression of several illnesses, including malignancies, heart failure, and neurological disorders (Onishi et al., 2021). Various studies have shown that mitophagy pathways are intimately associated with the metabolic rearrangement of cancer cells to meet the high bioenergetic needs of tumors (Vara-Perez et al., 2019). Although definitive functional changes and molecular mechanisms of mitophagy have not been completely elucidated, the expression levels of some mitophagy-related genes could serve as biomarkers for the diagnosis, prognosis, or therapy of some cancers (Wang et al., 2021; Wang et al., 2022). A recent paper revealed that mitophagy is closely related to the clinical prognosis of patients with UM, and indicated that mitophagy-related biomarkers (*PGAM5, SQSTM1, ATG9A*, and *GABARAPL1*) are survival-related genes of UM patients (Liu et al., 2022). However, the mitophagy-related genes that cause pathogenesis, as well as their importance in the diagnosis, prognosis, and therapeutic interventions of UM, remain unclear.

We screened and examined the mitophagy-related gene expression patterns of UM and normal samples by using an expression matrix of the samples from The Cancer Genome Atlas (TCGA, https://portal.gdc.cancer.gov/) and Gene Expression Omnibus (GEO, http://www.ncbi.nlm.nih.gov/geo) databases. We then constructed a mitophagy-related risk model and mitophagy score and performed functional analysis, clinical feature correlation analysis, and immune infiltration analysis. Correlation analysis of the clinical prognosis based on mitophagy-related gene scores was performed. Our findings provide unique insights into the critical roles that mitophagy plays in the development and prognosis of UM, as well as determining whether the mitophagy-related gene signature is a viable prognostic biomarker and potential treatment option for UM patients.

## Materials and methods

### Collecting data

Gene expression data of patients with UM were obtained from the TCGA (Hutter and Zenklusen, 2018) (https://portal.gdc.cancer.gov/) and GEO databases (Barrett et al., 2007) from the GSE22138 (Laurent et al., 2011) (https://www.ncbi.nlm.nih.gov/geo/query/acc.cgi?acc=GSE22138) dataset. The TCGA database contained the expression data and corresponding clinicopathological information for 80 UM samples, as shown in Table 1. The chip platform for the GSE22138 dataset (sample size:63) was the [HG-U133_Plus_2] Affymetrix Human Genome U133 Plus 2.0 Array, as shown in Table 1. The set of mitophagy-related genes was obtained from Wang et al, (2022).

**TABLE 1:** Baseline data table.

	TCGA	GSE22138
No. of patients	80	63
Age (median, range)	62 (22–86)	62 (28–84)
Gender (%)		
Female	35 (43.75%)	24 (38.10%)
Male	45 (56.25%)	39 (61.90%)

### UM patient cluster analysis using single-sample gene set enrichment analysis results

Bindea et al, (2013) determined that we have 23 gene sets relating to immune response. To assess the number of immune cells present in each sample, we measured the expression levels of genes that are unique to those cells. The R statistical environment (R core team) “GSVA” tool was used to run single-sample gene set enrichment analysis (ssGSEA). Patients’ immune cells were analyzed using the ssGSEA technique, and we used the “hclust” module of the “sparcl” program to separate UM patients into various groups based on the quantity of immune cells in each sample. The ESTIMATE Score, ImmuneScore, StromalScore, and tumor purity were proven using the “ESTIMATE” R program. This package was developed to assess the immune and stromal cell invasion of the tumor microenvironment (TME) using gene expression patterns; ImmuneScore represents the infiltration of immune cells in the tumor tissue; ESTIMATEScore is used to predict tumor purity, and StromalScore assesses the presence of stroma in tumor tissue. The expression of 23 immune cells in different groups was analysed using heat maps. Both datasets were then processed using the Perl programming language to identify mitophagy-related genes. Two immunological groups with wide variations in mitophagy-related gene expression were compared using principal component analysis (PCA), and the results were visualized using “ggplot2” to confirm if the grouping was legitimate. Mitophagy-related genes were subsequently identified using the R “limma” package (Ritchie et al., 2015), which was used to identify separate immunological groups of UM patients with a *p* < 0.05 screening cut-off.

### Construction of molecular isoforms of mitophagy-related genes

Depending on mitophagy-related gene expression, we performed co-expression analysis of mitophagy-related genes in UM and consistency clustering analysis of TCGA-UM data using the “ConsensusClusterPlus” (Wilkerson and Hayes, 2010) (http://www.bioconductor.org/packages/release/bioc/html/ConsensusClusterPlus.html)package in R. Samples were classified into different groups as shown by mitophagy-related gene expression, with parameters set to 50 replicates (reps = 50) and a resampling rate of 80% (pItem = 0.8). To determine the validity of the grouping, we performed PCA (UM) of the expression profiles of all genes, and the outcomes were displayed using the “ggplot2” package. In addition, we observed a correlation between the different groups and mitophagy-related genes.

### Gene set variation analysis and GSEA

Gene set variation analysis (GSVA) is a non-parametric unsupervised analytic approach primarily used to examine the microarray nuclear transcriptome gene set enrichment findings. We wanted to see if different metabolic pathways were more prevalent across samples by changing gene expression matrices to gene set matrices. The gene sets “c2. Kegg. v7.5.1″ and “c5.go. v7.5.1″ were retrieved from the MsigDB database (Liberzon et al., 2015) (http://www.gsea-msigdb.org/gsea/index.jsp). Utilizing the “GSVA” R package for GSVA enrichment analysis (Hänzelmann et al., 2013) and the “pheatmap” package for visualization, GSVA enrichment was carried out on two gene sets with distinct mitophagy-related gene molecular isoforms. Gene set enrichment analysis (GSEA) was used to evaluate the trend of the organization of genes from a preset gene set in a table of differentially expressed genes (DEGs) arranged by their phenotypic correlation, and thus to quantify their contribution to phenotype (Subramanian et al., 2005). The “c2. Kegg. v7.5.1″ and “c5.go. v7.5.1″ character gene sets were subjected to GSEA. The “clusterProfiler” R package (Yu et al., 2012) was used to accomplish GSEA evaluation, and a *p*-value of < 0.05 was regarded as statistically significant.

### Predictive model construction and validation

Utilizing genes linked with mitophagy, the prediction method was created. Based on the survival package threshold values, we initially classified all UM patients into high- and low-risk categories. Then, we used one-way COX regression analysis to identify crucial mitophagy-related genes and the “forestplot” R package to illustrate the results. Using the “glmnet” R package (Friedman et al., 2010), the training group was subjected to a least absolute shrinkage and choice operator (LASSO) regression analysis. The LASSO method reduces data size by employing a model with fewer components to describe the data attributes (Gui and Li, 2005). Using tenfold cross-validation, the training cohort-based model was prevented from overfitting. On the basis of the regression coefficients produced from the LASSO regression analysis, a suitable rating system and prognostic grouping were formed. To enhance the prediction accuracy of the model, which was developed by integrating clinical characteristics and prognostic risk scores to predict the probability of survival in patients with UM, we performed a receiver operating characteristic (ROC) curve analysis using the “survival” R package and measured the region under curves (AUCs) for various survival times. Using calibration curves and C-index readings generated from 1,000 rounds of bootstrap testing, the discriminatory power of column line graphs was evaluated. In addition, we examined the relationship between prognostic models and clinicopathological characteristics in the TCGA data. In addition, the expression of mitophagy-related genes was identified in the various risk groupings. Lastly, we employed the “survival” R package to examine the overall survival of the two patient groups.

### Immunopurity, immune infiltration, and functional enrichment analysis

To examine the link between these parameters, risk scores were first categorized by clinical features. Second, the “survival” package in R was employed to identify the impact of prognostic mitophagy-related gene expression on the survival of various risk groups. The link between the ESTIMATE score and high- and low-risk categories was investigated further.

The assessment of immune cell infiltration in UM patients using RNA-Seq data is a crucial tool for illness research, therapy prognosis prediction, and cellular infiltration estimation (Newman et al., 2019). We evaluated the link between immune cells and prognostic models to examine the link between the various models and the degree of immune infiltration. Lastly, we did GSVA analysis of “c2. Kegg. v7.5.1.” and “c5.go. v7.5.1.” symbol gene sets in various risk groups, performed GSVA enrichment analysis with the “GSVA” R package (Hänzelmann et al., 2013), and illustrated the results with the “pheatmap” package.

### Mitophagy score and immune cell correlation analysis

The R “PCA” package was applied to calculate the mitophagy level scores for each sample based on prognostic mitophagy-related gene expression. Subsequently, correlations between immune cells and mitophagy level scores were calculated to assess the relationship between different models and the level of immune infiltration.

### GEO dataset validation

Key mitophagy-related genes were identified by intersecting the gene lists produced from enrichment and correlation studies. We divided all GEO-UM patients into high- and low-risk sets based on the survival threshold levels provided by the R “survival” package. Then, a univariate COX regression analysis was carried out to screen for major impacts of mitophagy-related genes, and we displayed the results using the R “forestplot” package. Using the R “glmnet” package (Friedman et al., 2010), a LASSO regression analysis was then conducted on the training cohort. On the basis of the regression coefficients derived from LASSO regression analysis, a score system was developed, and prognostic grouping was done appropriately. To evaluate model stability, we performed ROC curve assessment with the “survival” package and produced AUCs for various survival periods. We studied the overall survival of patients in both groups using the R program “Kaplan–Meier survival” Furthermore, the expression of important genes in the various risk categories and groups with or without metastasis was evaluated.

### Statistical analysis

Using the R statistical environment and associated R packages (https://www.r-project.org/, version 4.0.2), all data were estimated and evaluated. The statistical accuracy of normally distributed values was assessed with an independent Student’s t-test, whilst differences between non-normally distributed parameters were examined with the Mann–Whitney *U* test (i.e., the Wilcoxon rank sum test). *p* < 0.05 was considered statistically significant for all two-sided *p*-values.

## Results

### Differential expression analysis of mitophagy-related genes in different immune groups

**FIGURE 1:**
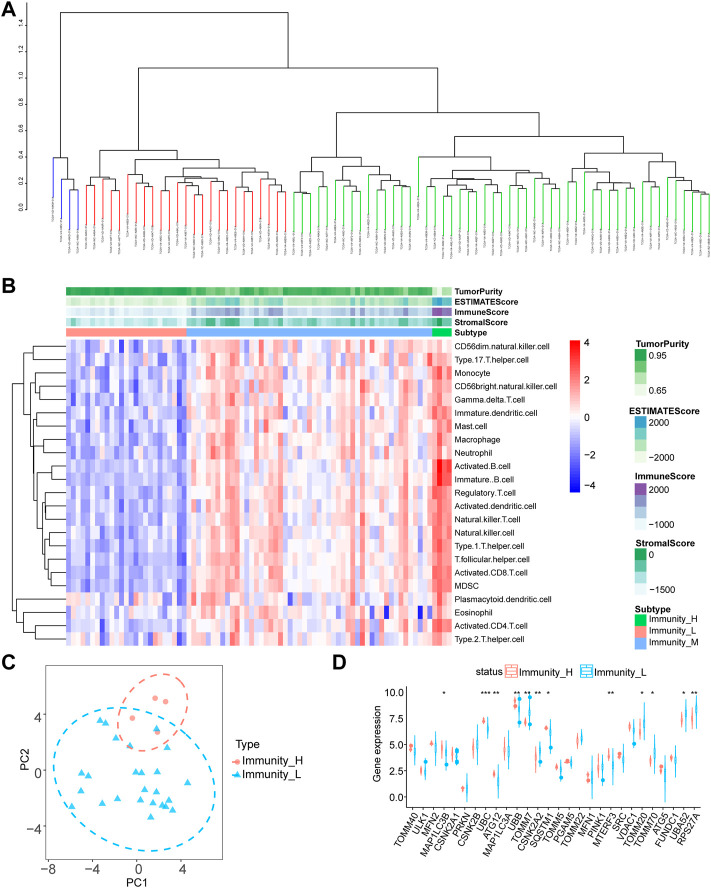
Differential expression analysis of mitophagy-related genes. **(A)** SsGSEA algorithm for hierarchical clustering of tumor samples in the TCGA-UM dataset was divided into three groups: high immune group, medium immune group and low immune group according to the degree of immune infiltration. **(B)** Complex heat map presentation of the results of immune infiltration analysis of immune subgroups of tumor samples from the TCGA-UM dataset. **(C)** The PCA analysis results of the high and low immune groups of tumor samples in the TCGA-UM data set are shown. **(D)** Group comparison graph showing the results of differential expression analysis of mitophagy-related genes between high and low immune subgroups of tumor samples in the TCGA-UM dataset, red represents the high immune group and blue represents the low immune group. Ns, not significant; *p < 0.05; **p < 0.01; ***p < 0.001. TCGA, The cancer genome atlas; UM, uveal melanoma; PCA, principal component analysis.

To evaluate the variability of mitophagy regulating genes in distinct immunological groups, we calculated the degree of infiltration of 23 immune cells in each sample using the ssGSEA method on TCGA-UM data and then used hierarchical clustering to separate the samples into three groups (Figure 1A). Green indicates the low immunity group (51 samples), blue the medium immunity group (4 samples), and red the high immunity group (25 samples). Using the ESTIMATE technique, we also determined the estimated score, immunological score, stromal score, and tumor purity for each sample. We utilized a heat map to illustrate the expression of immune cells in each sample subgroup, so we categorized the samples into high, medium, and low immune-level groups (Figure 1B). Consequently, we conducted a differential gene expression (DGE) study of mitophagy-related genes utilizing the two groups with the greatest differential expression (high and low immunity groups), and the PCA findings revealed that the high- and low-immunity groups had a greater efficiency of isolation (Figure 1C). Using the Wilcoxon test method, twelve genes were shown to be substantially differentially expressed across the high-and low-risk groups (Figure 1D; *p* < 0.001 for *UBC, p* < 0.01 for *ATG12, UBB, TOMM7, CSNK2A2, MTERF3, RPS27A, p* < 0.05 for *MAP1LC3B, SQSTM1, TOMM20, TOMM70,* and *UBA52*).

### Characterization of mitophagy-related gene expression in uveal melanoma

**FIGURE 2:**
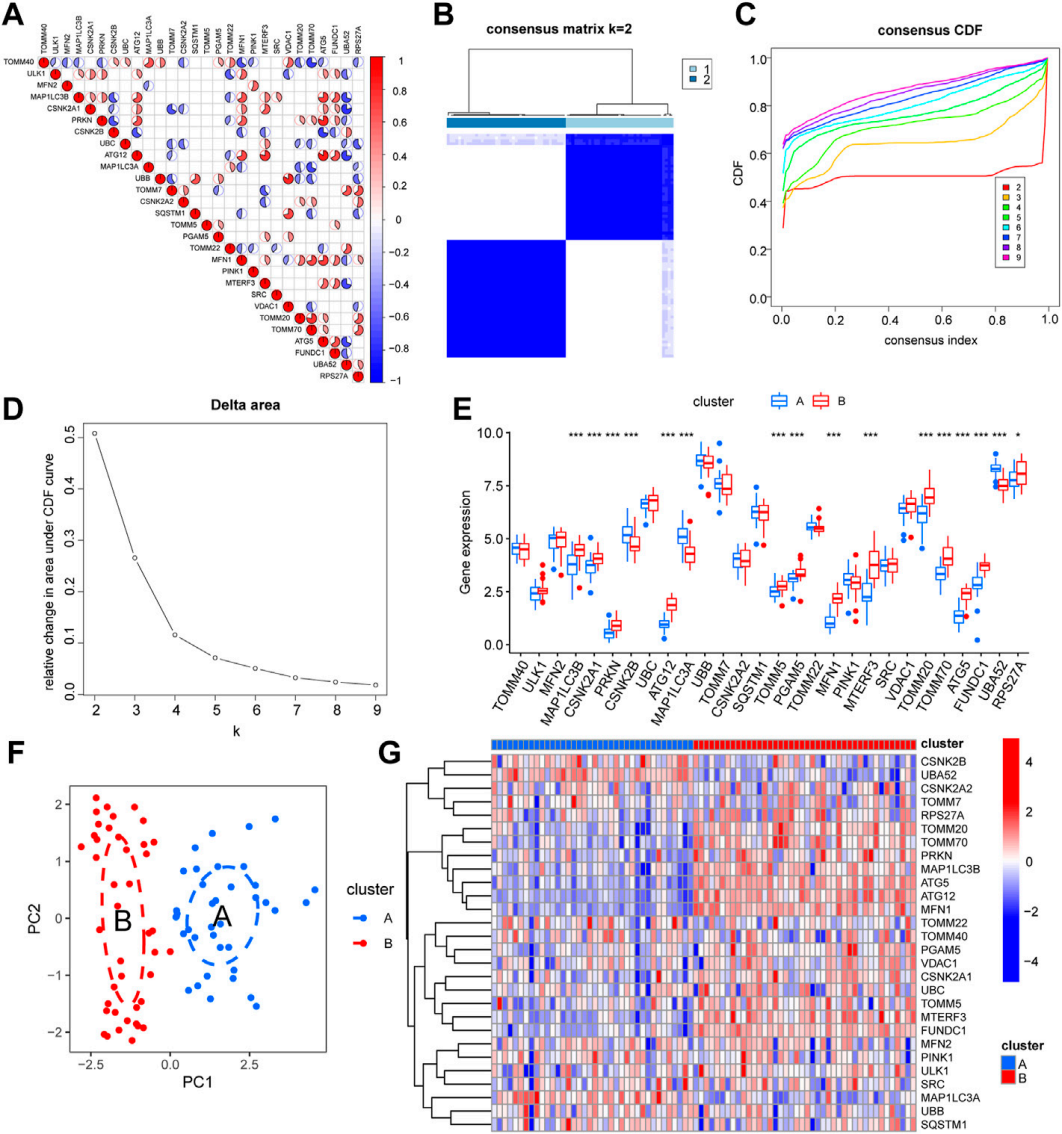
Expression characteristics and molecular grouping of mitophagy-related genes in uveal melanoma. **(A)** Correlation heat map display of mitophagy-related genes co-expression. **(B–D)** Consistent clustering (K = 2) results of TCGA-UM dataset based on mitophagy-related genes expression (B, cluster1: clusterA; cluster2: clusterB). CDF curves for different numbers of clusters in consistent clustering **(C)** and delta plot of area under the CDF curve **(D)**, dividing the samples into group A and group B. **(E)** Box plot of differential expression analysis of mitophagy-related genes in different isoforms of the TCGA-UM dataset, blue represents group A and red represents group B. **(F)** PCA analysis results of different subtypes of TCGA-UM dataset, blue represents group A, red represents group B. **(G)** Differential expression analysis of different isoforms of mitophagy-related genes in the TCGA-UM dataset complex heat map display, blue represents group A, red represents group B. Ns, not significant; *, *p* < 0.05; **, *p* < 0.01; ***, *p* < 0.001. TCGA, The cancer genome atlas; UM, uveal melanoma; CDF, cumulative distribution function; PCA, principal component analysis; KM, Kaplan–Meier.

We performed a co-expression study of mitophagy**-**related genes in UM/normal tissue to determine the influence of mitophagy**-**related genes on UM tissue (Figure 2A). The findings of the co-expression study demonstrated a substantial association between *MFN1* expression and that of many genes. To further explore the role of mitophagy-related genes in UM, we used the expression of mitophagy-related genes to perform hierarchical clustering of all TCGA samples with the parameter set to 50 replicates (reps = 50) and resampling rate of 80% (pItem = 0.8). All materials were categorized into two isoforms (A: *n* = 38; B: *n* = 42; Figures 2B–D), and the PCA findings demonstrated a high degree of separation (Figure 2F). Further differential expression assessment of mitophagy**-**related genes in groups A and B revealed that 16 genes were substantially differently expressed in distinct subgroups, with *p*-values of *ATG5, ATG12, CSNK2A1, CSNK2B, FUNDC1, MAP1LC3A, MAP1LC3B, MFN1, MTERF3, PGAM5, PRKN, TOMM5, TOMM20, TOMM70,* and *UBA5*2 being < 0.001 and *p*-values of *RPS27A* being < 0.05. (Figures 2E,G; Supplementary Figure S1).

### Evaluation of molecular isoform models of mitophagy-related genes

**FIGURE 3:**
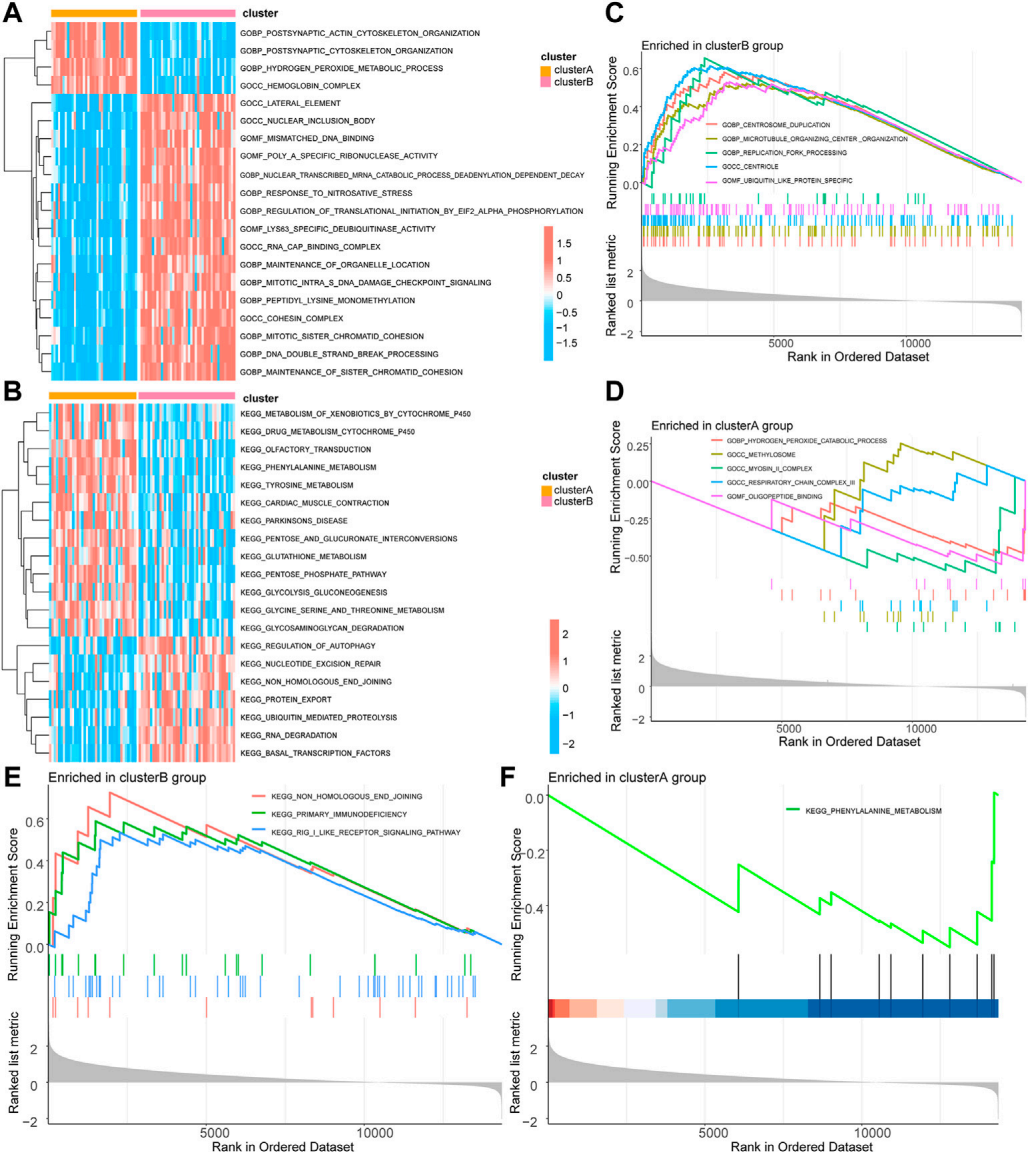
GSVA and GSEA of molecular subtypes of mitophagy-related genes. **(A)** GSVA-GO enrichment analysis heat map results based on the c5.go.v7.5.1.symbols gene set in different groups of the TCGA-UM dataset. **(B)** GSVA-KEGG enrichment analysis heat map results based on the c2.kegg.v7.5.1.symbols gene set in different groups of TCGA-UM dataset. **(C)** GSEA-GO cluster B enrichment analysis results pathway display. **(D)** GSEA-GO cluster A enrichment analysis results pathway display. **(E)** GSEA-KEGG cluster B enrichment analysis results pathway display. **(F)** GSEA-KEGG cluster A enrichment analysis results pathway display. GSVA, Gene Set Variation Analysis; TCGA, the cancer genome atlas; UM, uveal melanoma; GO, Gene Ontology; KEGG, Kyoto Encyclopedia of Genes and Genomes; GSEA, Gene Set Enrichment Analysis. The screening criteria for significant enrichment in GSEA enrichment analysis was *p* < 0.05.

We further performed GSVA based on different groups of molecular subtypes of mitophagy**-**related gene constructs. The results suggested that the cluster A group focused on functional association with centrosome duplication, microtubule organizing center organization, centriole, cul3 ring ubiquitin ligase complex, ciliary basal body replication fork processing, centriole assembly, ubiquitin-like protein-specific protease activity, RNA binding involved in post-transcriptional gene silencing, and DNA-binding transcriptional repressor activity (Figure 3; Supplementary Table S1).

### Construction of prognostic model of mitophagy-related genes and screening of key mitophagy-related genes

**FIGURE 4:**
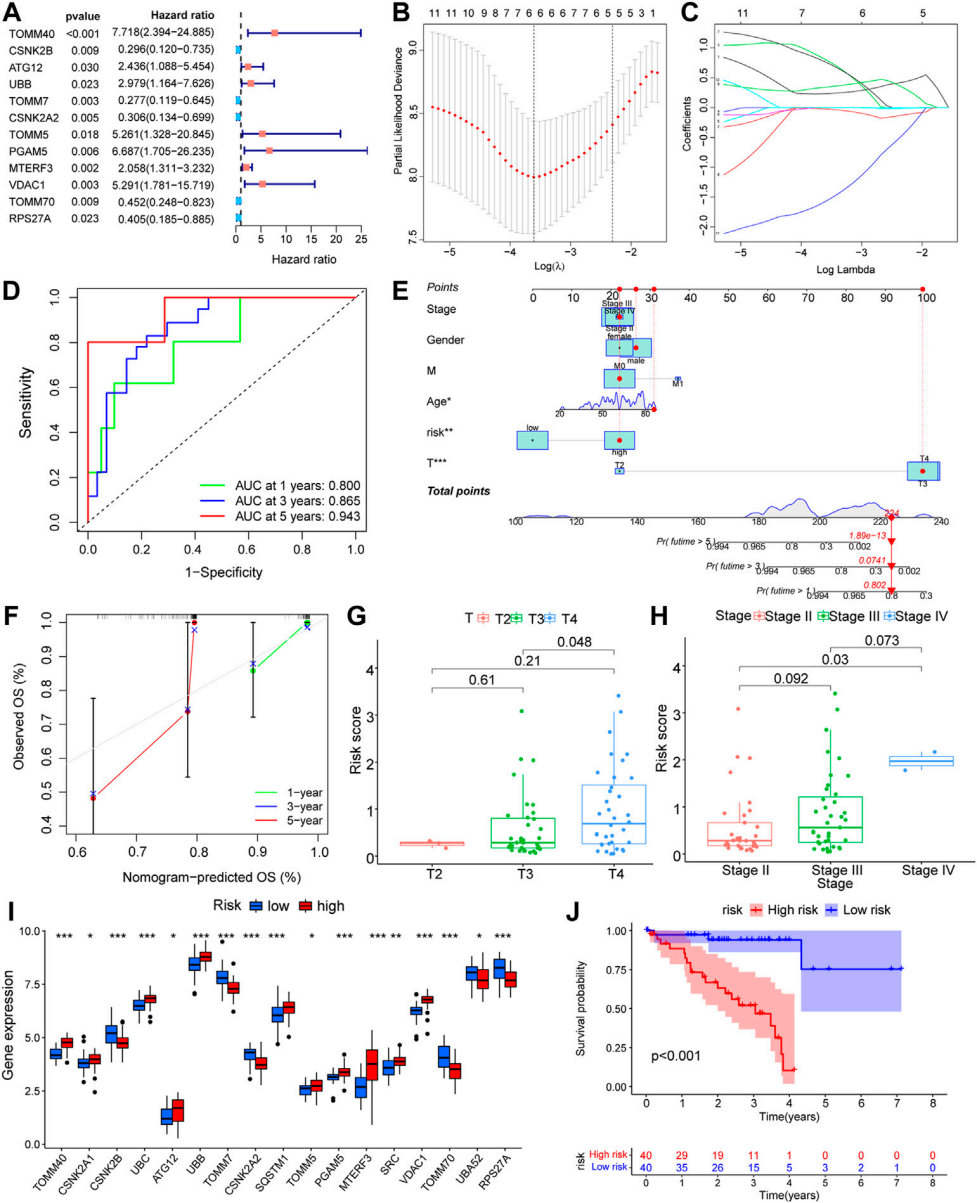
Prognostic model construction based on mitophagy-related genes. **(A)** Forest diagram showing the results of COX regression analysis based on mitophagy-related genes. **(B,C)** Prognostic model **(B)** and variable trajectory **(C)** of LASSO regression analysis model based on mitophagy-related genes. **(D)** Time-dependent ROC curve results of Riskscore of LASSO model are shown. Green is the AUC of patients who survived one year, blue is the AUC of patients who survived three years, and red is the AUC of patients who survived five years. **(E,F)** Nomogram results of different clinical variables combined with LASSO model Riskscore high and low groups **(E)** and results of 1-year, 3-year, 5-year calibration curve analysis **(F)**. **(G,H)** Difference analysis group comparison chart results of LASSO model Riskscore in different T stages **(G)** and different stage stages **(H)**. **(I)** The results of analysis of the differences in expression of mitophagy-related genes between high and low Riskscore groups in the LASSO model are shown in the group comparison chart, where blue is the low-risk group and red is the high-risk group. **(J)** KM curve results of survival analysis of the LASSO model with high and low Riskscore groups, blue is the low -risk group and red is the high-risk group. The closer the AUC in ROC curve was to 1, the better the diagnostic effect was. The AUC has low accuracy when it is between 0.5 and 0.7. The AUC has a certain accuracy between 0.7 and 0.9. The accuracy is higher when the AUC is above 0.9. Ns, not significant; *, *p* < 0.05; **, *p* < 0.01; ***, *p* < 0.001. LASSO, least absolute shrinkage and selection operator; KM, Kaplan–Meier; ROC, receiver operating characteristic.

To observe the effect of mitophagy**-**related genes on UM tissue, we performed one-way COX regression analysis of mitophagy**-**related genes in UM/normal tissue (Figure 4A; Table 2). Regression analysis identified 12 genes, including *TOMM40, CSNK2B, ATG12, UBB, TOMM7, CSNK2A2, TOMM5, PGAM5, MTERF3, VDAC1, TOMM70,* and *RPS27A*, which were significantly associated with UM; and a LASSO prognostic model containing six genes was constructed using significantly associated mitophagy-related gene expression in UM (Figures 4B–C). We multiply the gene expression in the TCGA-UM dataset of the LASSO prognostic model by the coefficient of each gene in the model, and then add them together to obtain the Riskscore of the LASSO prognostic model. The Riskscore calculation formula is as follows: $riskScore=\underset{i}{\sum }Coefficient\;\left({hub\;gene}_{i}\right)*mRNA\;Expression\;\left(hub\;{gene}_{i}\right)$

**TABLE 2:** Univariate COX analysis of TCGA data set.

id	HR	*p*-value
TOMM40	7.718	< 0.001
MTERF3	2.058	< 0.01
VDAC1	5.291	< 0.01
TOMM7	0.277	< 0.01
CSNK2A2	0.306	< 0.01
PGAM5	6.687	< 0.01
CSNK2B	0.296	< 0.01
TOMM70	0.452	< 0.01
TOMM5	5.261	< 0.05
UBB	2.979	< 0.05
RPS27A	0.405	< 0.05
ATG12	2.436	< 0.05

The Riskscore calculated according to the LASSO prognostic model was segmented into high- and low-risk groups depending on the median and then verified using AUC for the prognostic model, revealing that the risk level had good predictive accuracy for 1-, 3-, and 5-year patient survival (Figure 4D). In addition, we developed a nomogram (Figure 4E) and a calibration plot (Figure 4F) by combining the risk score of the LASSO model with clinical features, such as patient age, gender, and TNM stage. In addition, we obtained the clinical data of the samples to link several clinical parameters with the LASSO risk score. The findings indicate that both the T-stage and risk score of the tumor had a substantial impact on the prognosis. We also found that sample risk score rose substantially with increasing T-stage and grade (Figures 4G,H), which was consistent with our earlier assumption. The majority of mitophagy-related genes were substantially differently expressed within subgroups (Figure 4I), where the *p*-values of *TOMM40, CSNK2B, UBC, UBB, TOMM7, CSNK2A2, SQSTM1, PGAM5, MTERF3, VDAC1, TOMM70*, and *RPS27A* were < 0.001, the *p*-value of *SRC* was < 0.01, and the *p*-values of *CSNK2A1*, *ATG12*, *TOMM5*, and *UBA52* were < 0.05. In conclusion, the survival analysis of the high-risk and low-risk groups revealed that the low-risk group had greater survival rates (Figure 4J).

**FIGURE 5:**
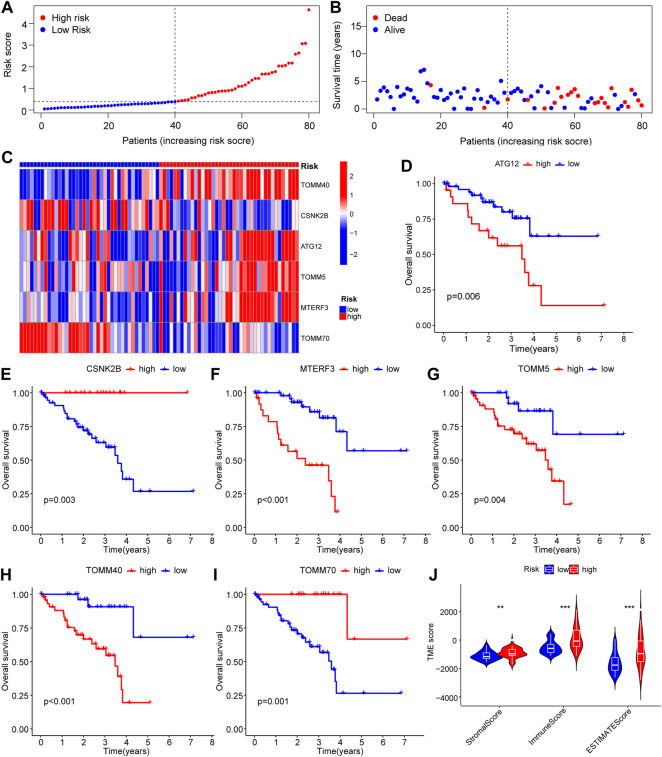
Survival-related analysis based on the prognostic model of mitophagy-related genes. **(A–C)** Riskscore of patient samples between high and low Riskscore groups in LASSO model **(A)** and expression of survival status of patients between high and low risk groups **(B)** and complex heat map results of prognostic genes in the model **(C)**. **(D–I)** KM curves for survival analysis of mitophagy-related genes, including ATG12 **(D)**, *CSNK2B* **(E)**, *MTERF3* **(F)**, *TOMM5* **(G)**, *TOMM40* **(H)** and *TOMM70* **(I)** in the LASSO model. **(J)** Estimate analysis results between high and low Riskscore groups of the LASSO model show the group comparison of Stromal Score, Immune Score, and ESTIMATE Score. Ns, not significant; *, *p* < 0.05; **, *p* < 0.01; ***, *p* < 0.001. LASSO, least absolute shrinkage and selection operator; KM, Kaplan–Meier.

Investigation of the LASSO outcome demonstrated that patients with high-risk scores had a reduced rate of survival and a higher mortality rate (Figures 5A–C). Survival study of the screened core genes reported a significant association between the expression of *ATG12, CSNK2B, MTERF3, TOMM5, TOMM40*, and *TOMM70* with patient survival rate (Figures 5D–I). The findings of the ESTIMATE data show that samples from patients with high-risk scores had a greater ESTIMATE score (Figure 5J).

### Correlation analysis between risk score and immune cell infiltration

**FIGURE 6:**
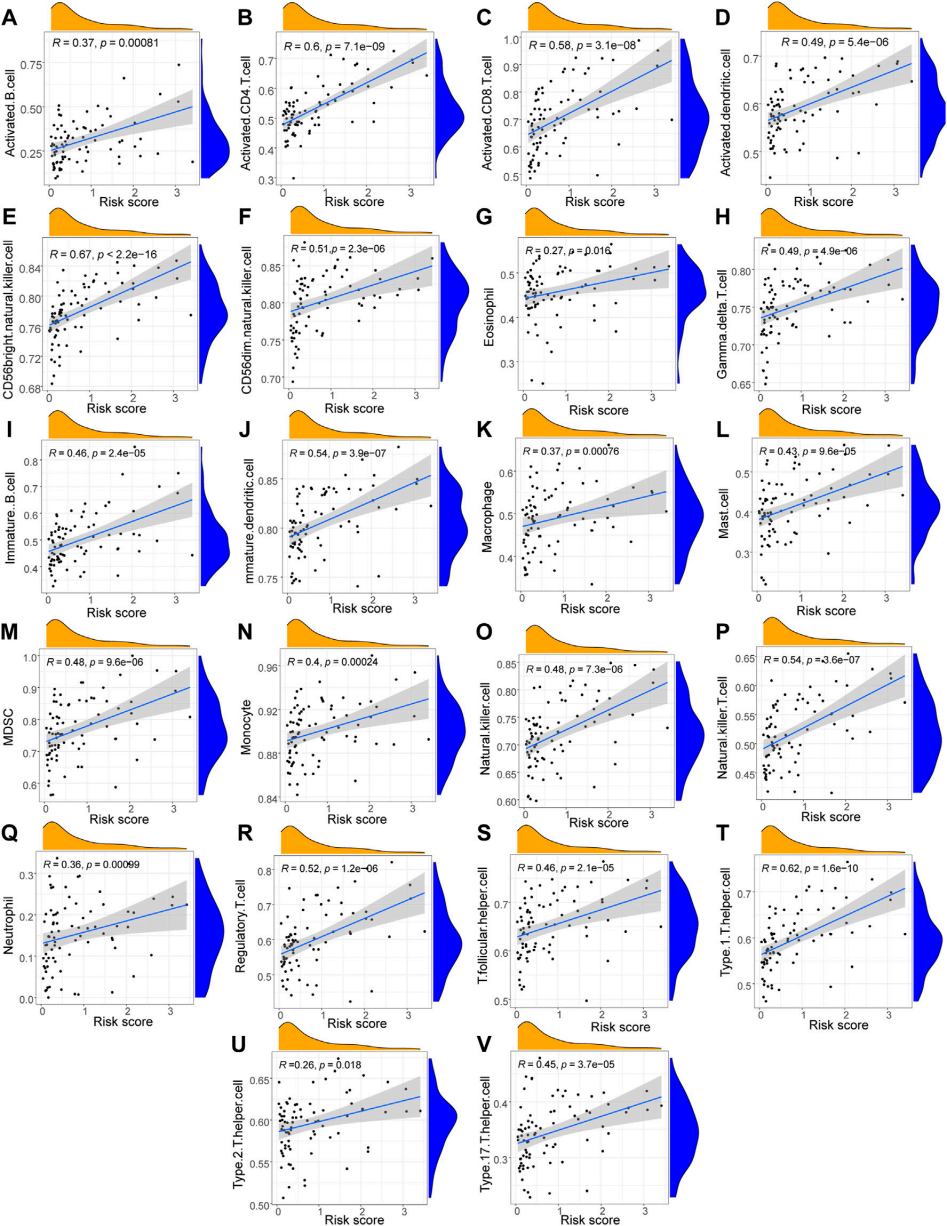
Correlation analysis between immune cells and Riskscores. **(A–V)** The scatter plot of the correlation analysis results between the LASSO model risk score and the infiltration abundance of immune cell including Activated B cells **(A)**, Activated CD4 T cells **(B)**, Activated CD8 T cells **(C)**, Activated dendritic cells **(D)**, CD56 bright natural killer cells **(E)**, CD56dim natural killer cells **(F)**, Eosinophils **(G)**, Gamma delta T cells **(H)**, Immune B cells **(I)**, Mature dendritic cells **(J)**, Macrophages **(K)**, Mast cells **(L)**, MDSCs **(M)**, Monocytes **(N)**, Natural killer cells **(O)**, Natural killer T cells **(P)**, Neutrophils **(Q)**, Regulatory T cells **(R)**, T follicular helper cells **(S)**, Type1 T helper cells **(T)**, Type2 T helper cells **(U)**, Type 17 T helper cells **(V)**. The slope is the magnitude of the correlation, and the *p*-value indicates the level of significance. *p* ≥ 0.05, not statistically significant; *p* < 0.05, statistically significant; *p* < 0.01, highly statistically significant; *p* < 0.001, extremely statistically significant. The absolute value of the correlation coefficient (R) in the correlation scatter plot is above 0.8, which is a strong correlation; The absolute value between 0.5 and 0.8 is a moderate degree of correlation; The absolute value between 0.3 and 0.5 is weakly correlated; The absolute value below 0.3 is considered weak or uncorrelated.

Using the Wilcoxon test method, we identified 22 immune cells that were substantially less penetrated in the high-risk group compared with the low-risk group (Figure 6). The cells consisted of activated B cells, activated CD4^+^ T cells, activated CD8^+^ T cells, activated dendritic cells, CD56 bright natural killer cells, CD56 dim natural killer cells, eosinophils, gamma delta T cells, immune B cells, mature dendritic cells, macrophages, mast cells, MDSCs, monocytes, natural killer cells, natural killer T cells, neutrophils, regulatory T cells, T follicular Eosinophils, and Type 2 T helper cells, which had *p* values of < 0.01; the remaining 20 immune cells had *p* values of < 0.001.

**FIGURE 7:**
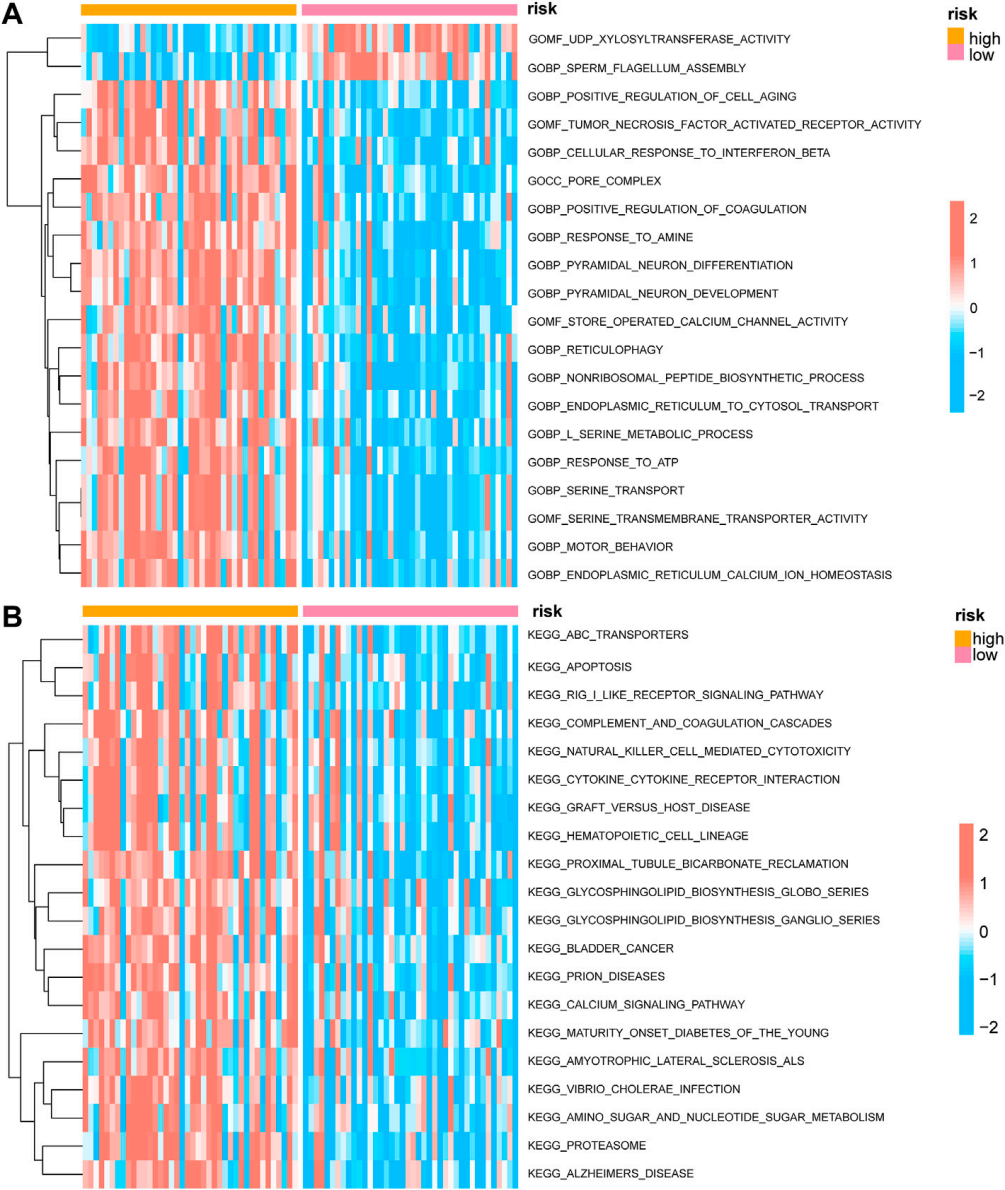
GSVA enrichment analysis between high and low prognostic risk groups. **(A)** Complex heat map showing the results of GSVA-GO analysis between low and high prognostic risk groups. **(B)** Complex heat map showing the results of GSVA-KEGG analysis between low and high prognostic risk groups. GSVA, gene set variation analysis; GO, gene ontology; KEGG, Kyoto Encyclopedia of Genes and Genomes.

We conducted a GSVA assessment of the molecular functions of the various groups based on the immunological score construction model classification of high-and low-risk groups. The gene ontology (GO) results showed that the low-risk group was concerned with activities linked to UDP-xylosyltransferase activity, sperm flagellum construction, positive control of cell ageing, tumor necrosis factor-activated receptor activity, and cellular response to interferon beta (Figure 7A). The results of the Kyoto Encyclopedia of Genes and Genomes (KEGG) indicated that the low-risk group focused on correlating with ABC transporters, apoptosis, RIG-I-like receptor signaling mechanism, complement and coagulation cascades, natural killer cell-mediated cytotoxicity, cytokine-cytokine receptor interaction, graft *versus* host disease, and other functions (Figure 7B).

### Mitophagy score and immune cell correlation analysis

We used the PCA technique to score mitophagy levels in individual samples to obtain the Mitophagy Score in order to investigate the link between mitophagy and the presence of immune cells in patients with uveal melanoma. From the Wilcoxon results, it was determined that 10 immune cells were substantially different from the mitophagy level score (Supplementary Figure S2); these included CD56 dim natural killer cells, eosinophils, gamma delta T cells, mature B cells, macrophages, mast cells, MDSCs, monocytes, neutrophils, and type 2 T helper cells. Five immune cells had *p* < 0.01 and four immune cells displayed *p* < 0.01.

### GEO dataset validation

**FIGURE 8:**
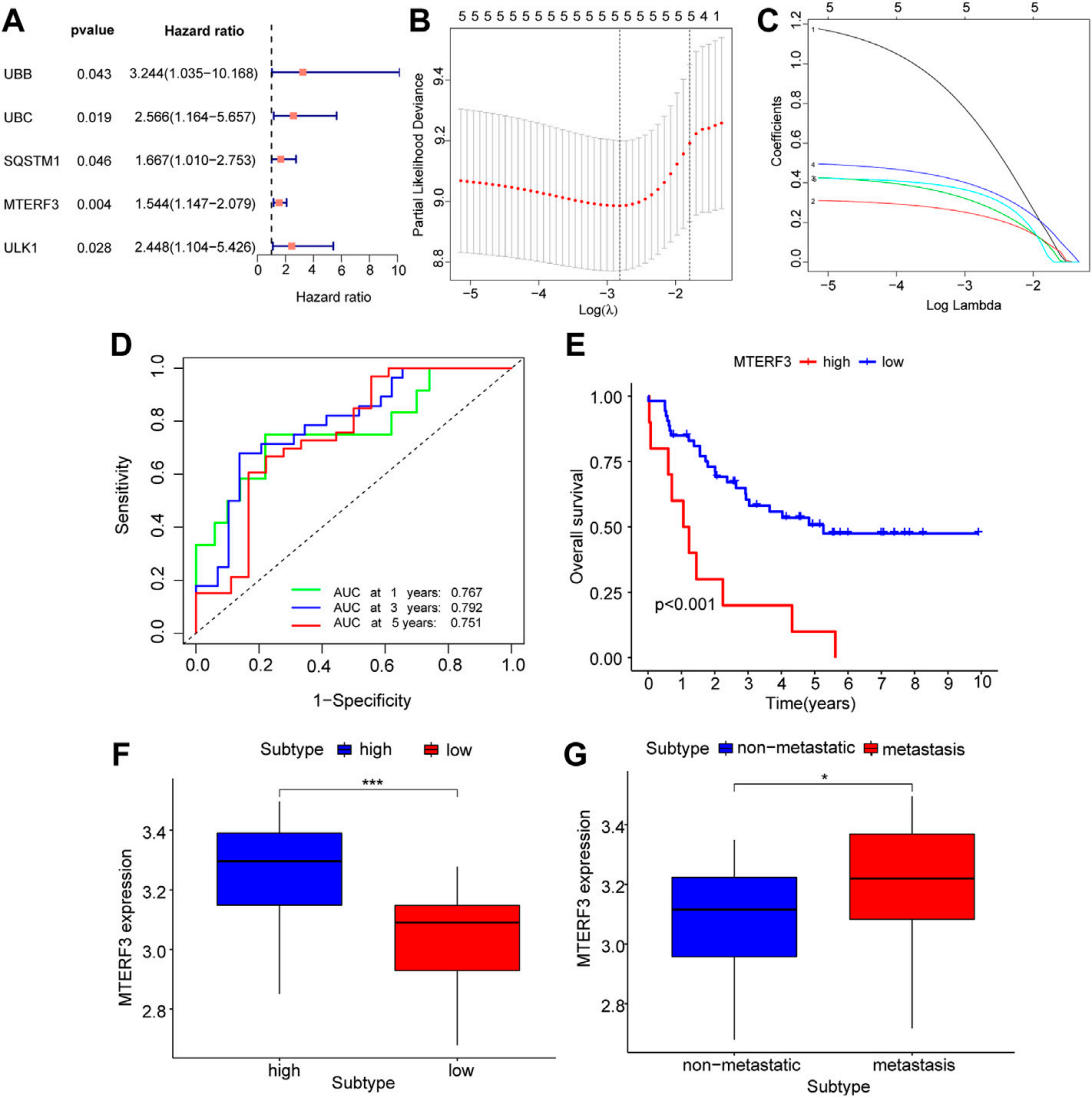
GEO dataset validates key mitophagy-related genes. **(A)** Forest diagram showing the results of COX regression analysis of mitophagy-related genes based on the GEO dataset. **(B,C)** Prognosis model diagram **(B)** and variable trajectory diagram **(C)** of LASSO regression analysis model based on the GEO dataset of mitophagy-related genes. **(D)** The time-dependent ROC curve results of Riskscore of the LASSO model based on the GEO dataset, green is the AUC of patients who survived for one year, blue is the AUC of patients who survived for three years, and red is the AUC of patients who survived for five years. **(E)** KM curve results of survival analysis of the LASSO model with high and low Riskscore groups, show that blue is the low-risk group and red is the high-risk group. **(F–G)** Based on the GEO dataset, the results of group comparison between the high and low Riskscore groups **(F)** and whether to transfer group **(G)** of mitophagy-related gene MTERF3 in the LASSO model. High risk group in blue, low risk group in red **(F)**; blue represents the non-metastasized group, red represents the metastasized group **(G)**. The closer the AUC in ROC curve was to 1, the better the diagnostic effect was. The AUC has low accuracy when it is between 0.5 and 0.7. The AUC has a certain accuracy between 0.7 and 0.9. The accuracy is higher when the AUC is above 0.9. Ns, not significant; *, *p* < 0.05; **, *p* < 0.01; ***, *p* < 0.001. LASSO, least absolute shrinkage and selection operator; KM, Kaplan–Meier; ROC, receiver operating characteristic.

DEGs from different immune groups, different molecular subtype groups, and prognostic genes obtained from LASSO regression screening were overlapped to identify key mitophagy-related genes, including *MTERF3*, *TOMM70*, and *ATG12*. Additional validation using the GEO dataset was conducted by splitting all GEO-UM patients into high- and low-risk groups based on survival analysis critical values, and then performing one-way COX regression (Figure 8A; Table 3) and LASSO regression analysis (Figures 8B–C) to create a prognostic model grouping that included *UBB, UBC, SQSTM1, MTERF3,* and *ULK1*. To evaluate model stability, further ROC curve analysis was undertaken to estimate the AUC for various survival periods; the findings indicated that the model was stable (Figure 8D). Survival study demonstrated the solid predictive character of the model (Figure 8E). The important mitophagy-related genes were intersected with the prognostic genes from GEO to identify the hub gene *MTERF3*, and the expression difference of the key *MTERF3* gene was confirmed in various GEO risk (Figure 8F) and metastatic groups (Figure 8G). *MTERF3* was substantially expressed in both high-risk and metastatic groups; hence, we hypothesized that *MTERF3* may be employed as a cancer-promoting gene in uveal melanoma.

**TABLE 3:** Univariate COX analysis of GEO data set.

id	HR	*p*-value
MTERF3	1.54	< 0.01
UBC	2.57	< 0.05
ULK1	2.45	< 0.05
UBB	3.24	< 0.05
SQSTM1	1.67	< 0.05

## Discussion

In our research, we examined the impact of mitophagy in UM. Differential expression analysis of mitophagy**-**related genes in various immunological groups revealed 12 DEGs in the high- and low-risk groups. Later, TCGA samples were classified into two subtypes using hierarchical clustering analysis based on the expression of mitophagy-related genes, and 16 DEGs were discovered in separate subtypes. A recent paper revealed that mitophagy-related biomarkers (*PGAM5, SQSTM1, ATG9A*, and *GABARAPL1*) were survival-related genes of UM patients, which could be used to predict the survival of these patients (Liu et al., 2022). Our results also showed the high expression of *PGAM5* and *SQSTM1* in the high immune-level and high-risk groups. Another study further validated that the high expression of *SQSTM1* was a high-risk factor for the lowest survival rate of UM patients (Fei et al., 2022). Given this, our results are reproducible and reliable and the mitophagy-related role of *PGAM5* and *SQSTM1* are worth being investigated in future studies.

The results of the GSEA demonstrated that the various mitophagy subtypes may be engaged in signaling pathways linked to cancer, mitochondrial metabolism, and modulatory signaling. Cancer cell proliferation, abnormal mitochondrial metabolism, and a remodeled tumor immune microenvironment are associated with UM growth and metastasis (Castet et al., 2019; Giallongo et al., 2020; Garcia-Mulero et al., 2021), consistent with our results. Enrichment analysis results confirmed the validity of the mitophagy**-**related genes identified in our study. Thus, we speculate that the differentially expressed mitophagy**-**related genes play key roles in facilitating the poor prognosis of UM, possibly by regulating cancer cell proliferation and immune or TME factors. A mitophagy-related risk model and nomogram were constructed using LASSO and Cox regression analysis to anticipate the prognosis of UM. A unique prognostic pattern consisting of six mitophagy-related genes was identified as a crucial independent prognostic factor for predicting the long-term prognosis of UM patients. The risk scores of mitophagy-associated signatures were connected to survival rate, tumor stage, and T stage, according to a correlation study. Depending on the risk model, patients were categorized as high- or low-risk. Owing to the poorer prognosis of high-risk patients, more aggressive therapies and shorter follow-up periods are necessary, suggesting that this risk model might help in the provision of accurate and tailored therapy in clinical practice.

Six key mitophagy-related genes (*ATG12, CSNK2B, MTERF3, TOMM5, TOMM40,* and *TOMM70*) were screened. *ATG12* is a human homolog of the yeast protein involved in autophagy (Murrow and Debnath, 2015). One study reported that the overexpression of lncRNA ZNNT1 can promote *ATG12*-dependent cell death to inhibit UM tumor cell growth and migration (Li et al., 2020a). In contrast, different research discovered frameshift mutations with single nucleotide repeats in *ATG12* genes in gastric and colorectal tumors, which may contribute to the advancement of cancer by dysregulating the autophagy process (Li et al., 2020b). C*SNK2B* encodes the beta component of casein kinase II, a protein that controls physiological mechanisms, signal transduction, transcription, translation, and replication (Heller-Harrison et al., 1989). Overexpression of *CSNK2B* in hepatocellular carcinoma and colorectal cancer increases cell division and prevents death (Yu et al., 2021). Mitochondrial transcription is inhibited by *MTERF3* [20]. High *MTERF3* expression correlates with cancer development and predicts a poor outcome in brain glioma patients (Zi et al., 2019). Interestingly, our findings also revealed that *MTERF3* might be exploited as a cancer-promoting gene in UM, which is associated with illness advancement and poor prognosis. *TOMM5, TOMM40*, and *TOMM70* are elements of the mitochondrial outer membrane translocase complex (Melin et al., 2014; Wang et al., 2020; Pitt and Buchanan, 2021), which facilitates the supply of proteins into mitochondria. P53-tom5 slows the proliferation of human A549 non-small cell lung cancer cells *via* directly impairing mitochondria (Umemoto et al., 2011). By controlling mitochondrial activity and increasing cellular energy and redox state, *TOM40* supports the formation of epithelial ovarian cancer; greater levels of *TOM40* protein expression are linked to fewer survival outcomes (Yang et al., 2020). Richter et al. identified *TOM70* as the primary target of RL2 in the mitochondrial membrane and proved that RL2 acts primarily on the mitochondria, causing reduced ATP generation and apoptosis in breast cancer (Richter et al., 2020). Autophagy can serve a balanced, tumor-inhibiting, or tumor-promoting function in multiple circumstances and phases of malignant transformation, depending on the cancer type, milieu, pathogenic circumstances, existence and state of the immune system, and stage of cancer progression (Li et al., 2020b). The mechanism of these six UM genes is not yet completely understood. Further investigation of their roles in UM pathogenesis, particularly in relation to mitophagy, may generate new lines of inquiry.

Another important result of our research is that the mitophagy score is linked to the level of CD56-dim natural killer cells, eosinophils, gamma-delta T cells, mature B cells, macrophages, mast cells, MDSCs, monocytes, neutrophils, and type 2 T helper cells. Several studies (Coussens and Werb, 2002; Horii and Matsushita, 2021) have found that the release of extracellular proteases, proangiogenic agents, and chemokines by macrophages, neutrophils, mast cells, eosinophils, and activated T lymphocytes promotes tumor development. Furthermore, immune cell invasion may influence the response to cancer immunotherapy (Zhang and Zhang, 2020). Thus, we believe that dysregulated mitophagy-related gene-related mechanisms may influence the immunotherapy response in UM. There is a need for additional research into the possible mechanisms of the mitophagy-related gene signature, mitophagy-related hazard ratio, and infiltrating immune cells, which may provide potential assistance for personalized therapies and possibilities for the creation of fresh treatment interventions for UM. In conclusion, our findings suggest that mitophagy-related genes may influence the tumor immune milieu and the prognosis of UM.

Our integrated microarray and RNA-seq data analysis showed for the first time that mitophagy-related signature genes are implicated in UM progression, prognosis, and TME immune cell infiltration. However, our study had certain restrictions. All study data were obtained from a publicly accessible database, which may contain biases based on race, location, and/or other demographic characteristics. What’s more, the findings were derived from bioinformatics analysis and lacked experimental confirmation using solid clinical specimens. It is necessary to accumulate UM samples for sequencing and further expand the sample size to validate our findings. Experiments such as western blot (WB), quantitative real-time PCR, and immunohistochemistry analysis are available to examine the primary expression of these predicted mitophagy-related genes in clinical tissue samples associated with UM. Furthermore, to elucidate the function of mitophagy-related genes in UM, studies on loss-of-function and gain-of-function at the molecular, cellular and biological levels are essential. Relevant molecular experiments may provide detailed and robust evidence for these predictive genes regulatory pathways in UM. In addition, the incorporation of many datasets in our study may have resulted in batch-to-batch discrepancies that could not be prevented or eliminated during evaluation. Finally, this unique mitophagy-related risk model has not been extended to all cancer types, which is worth being investigated in future studies.

## Conclusion

We developed a unique mitophagy-related risk model that offers a potential viable prognostic predictor for UM. The expression of mitophagy-related signature genes was also linked with the tumor milieu and immune cell infiltration, as shown by additional research into the molecular processes of mitophagy. Therefore, this research demonstrates the importance of mitophagy in UM, which may provide unique and promising indicators for the accurate prediction of clinical outcomes and choice of customized therapy targets. Additional *in vitro* and *in vivo* research is necessary to confirm our findings.

## Data Availability

The original contributions presented in the study are included in the article/Supplementary Material, further inquiries can be directed to the corresponding author.

## Supplementary material

SUPPLEMENTARY FIGURE S1 Analysis of mitophagy-related genes expression among different subgroups. Group comparison diagram showing the results of differential expression analysis of mitophagy-related genes in different groups of TCGA-UM dataset, including **(A)** *ATG5*. **(B)** *ATG12*. **(C)** *CSNK2A1*. **(D)** *CSNK2B*. **(E)** *FUNDC1*. **(F)** *MAP1LC3A*. **(G)** *MAP1LC3B*. **(H)** *MFN1*. **(I)** *MTERF3*. **(J)** *PGAM5*. **(K)** *PRKN*. **(L)** *RPS27A*. **(M)** *TOMM5*. **(N)** *TOMM20*. **(O)** *TOMM70*. **(P)** *UBA52*. Ns, not significant; **p* < 0.05; ***p* < 0.01; ****p* < 0.001. TCGA, the cancer genome atlas; UM, uveal melanoma.

SUPPLEMENTARY FIGURE S2 Correlation analysis between immune cells and mitophagy-related genes scores. The scatter plot of the correlation analysis results between mitophagy-related genes score and the infiltration abundance of immune cell including CD56dim.natural.killer.cells **(A)**, Eosinophils **(B)**, Gamma delta T cells **(C)**, Mature B cells **(D)**, Macrophages **(E)**, Mast cells **(F)**, MDSCs **(G)**, Monocytes **(H)**, Neutrophils **(I)** and Type 2 T helper cells **(J)**. The slope is the magnitude of the correlation, and the p-value indicates the level of significance. *P* ≥ 0.05, not statistically significant; *P* < 0.05, statistically significant; *P* < 0.01, highly statistically significant; *P* < 0.001, extremely statistically significant. The absolute value of the correlation coefficient (R) in the correlation scatter plot is above 0.8, which is a strong correlation; The absolute value between 0.5 and 0.8 is a moderate degree of correlation; The absolute value between 0.3 and 0.5 is weakly correlated; The absolute value below 0.3 is considered weak or uncorrelated.
