# Supplementary material for: Integrative analyses of a mitophagy-related gene signature for predicting prognosis in patients with uveal melanoma
Source: Front Genet. 2022 Dec 5;13:1050341. doi: 10.3389/fgene.2022.1050341 (PMC9760814; doi:10.3389/fgene.2022.1050341)
Supplement: Supplementary file 1 [file Table1.docx]

Table S1 GSEA analysis of different mitophagy gene models

| **Description** | **Enrichment Score** | **NES** | **p.adjust** |
| --- | --- | --- | --- |
| GOBP_CENTROSOME_DUPLICATION | 0.58 | 1.41 | 9.99E-04 |
| GOBP_MICROTUBULE_ORGANIZING_CENTER_ORGANIZATION | 0.52 | 1.31 | 9.99E-04 |
| GOBP_REPLICATION_FORK_PROCESSING | 0.65 | 1.52 | 1.00E-03 |
| GOBP_CENTRIOLE_ASSEMBLY | 0.63 | 1.49 | 1.00E-03 |
| GOBP_DNA_DEPENDENT_DNA_REPLICATION_MAINTENANCE_OF_FIDELITY | 0.63 | 1.48 | 1.00E-03 |
| GOBP_CORTICOSTEROID_RECEPTOR_SIGNALING_PATHWAY | 0.78 | 1.65 | 1.04E-03 |
| GOBP_ADAPTIVE_IMMUNE_RESPONSE | 0.47 | 1.20 | 2.00E-03 |
| GOBP_CILIUM_ORGANIZATION | 0.49 | 1.26 | 2.00E-03 |
| GOBP_PROTEIN_LOCALIZATION_TO_MICROTUBULE_ORGANIZING_CENTER | 0.62 | 1.45 | 3.00E-03 |
| GOBP_REGULATION_OF_ESTABLISHMENT_OR_MAINTENANCE_OF_CELL_POLARITY | 0.70 | 1.56 | 3.03E-03 |
| GOBP_PHOSPHATIDYLINOSITOL_PHOSPHATE_BIOSYNTHETIC_PROCESS | 0.55 | 1.33 | 4.00E-03 |
| GOBP_CELL_PROJECTION_ASSEMBLY | 0.45 | 1.16 | 5.99E-03 |
| GOBP_PROTEIN_K48_LINKED_DEUBIQUITINATION | 0.62 | 1.43 | 6.00E-03 |
| GOBP_HISTONE_MRNA_CATABOLIC_PROCESS | 0.70 | 1.52 | 6.13E-03 |
| GOBP_NEGATIVE_REGULATION_OF_UBIQUITIN_PROTEIN_LIGASE_ACTIVITY | 0.74 | 1.53 | 6.27E-03 |
| GOBP_NON_MOTILE_CILIUM_ASSEMBLY | 0.56 | 1.36 | 6.99E-03 |
| GOBP_PROTEIN_MODIFICATION_BY_SMALL_PROTEIN_REMOVAL | 0.49 | 1.22 | 6.99E-03 |
| GOBP_DNA_REPAIR | 0.44 | 1.15 | 7.99E-03 |
| GOBP_NEGATIVE_REGULATION_OF_DNA_METABOLIC_PROCESS | 0.52 | 1.29 | 7.99E-03 |
| GOBP_NEGATIVE_REGULATION_OF_LEUKOCYTE_PROLIFERATION | 0.54 | 1.31 | 7.99E-03 |
| GOBP_NEGATIVE_REGULATION_OF_TELOMERE_MAINTENANCE | 0.61 | 1.40 | 9.00E-03 |
| GOBP_CENTRIOLE_CENTRIOLE_COHESION | 0.70 | 1.48 | 9.38E-03 |
| GOBP_REGULATION_OF_ENDOTHELIAL_CELL_DEVELOPMENT | 0.70 | 1.48 | 9.38E-03 |
| GOBP_NEGATIVE_REGULATION_OF_CILIUM_ASSEMBLY | 0.70 | 1.48 | 1.03E-02 |
| GOBP_REGULATION_OF_PROTEIN_GLYCOSYLATION | 0.74 | 1.51 | 1.06E-02 |
| GOBP_CELLULAR_RESPONSE_TO_INTERFERON_GAMMA | 0.52 | 1.28 | 1.10E-02 |
| GOBP_CONSTITUTIVE_HETEROCHROMATIN_ASSEMBLY | 0.74 | 1.50 | 1.17E-02 |
| GOBP_MICROTUBULE_CYTOSKELETON_ORGANIZATION | 0.44 | 1.13 | 1.20E-02 |
| GOBP_RESPONSE_TO_INTERFERON_GAMMA | 0.51 | 1.25 | 1.20E-02 |
| GOBP_REGULATION_OF_CENTRIOLE_REPLICATION | 0.65 | 1.44 | 1.22E-02 |
| GOBP_SERTOLI_CELL_DIFFERENTIATION | 0.70 | 1.47 | 1.26E-02 |
| GOBP_REGULATION_OF_CENTROSOME_CYCLE | 0.56 | 1.32 | 1.30E-02 |
| GOBP_RESPONSE_TO_X_RAY | 0.61 | 1.39 | 1.30E-02 |
| GOBP_EYE_PHOTORECEPTOR_CELL_DIFFERENTIATION | 0.68 | 1.49 | 1.32E-02 |
| GOBP_MITOTIC_G2_DNA_DAMAGE_CHECKPOINT_SIGNALING | 0.58 | 1.36 | 1.40E-02 |
| GOBP_PROGRAMMED_NECROTIC_CELL_DEATH | 0.58 | 1.37 | 1.40E-02 |
| GOBP_REGULATION_OF_TRANSCRIPTION_BY_RNA_POLYMERASE_III | 0.60 | 1.37 | 1.50E-02 |
| GOBP_NEGATIVE_REGULATION_OF_CHROMOSOME_ORGANIZATION | 0.51 | 1.26 | 1.60E-02 |
| GOBP_TELOMERE_MAINTENANCE_IN_RESPONSE_TO_DNA_DAMAGE | 0.69 | 1.44 | 1.88E-02 |
| GOBP_PROTEIN_LOCALIZATION_TO_CHROMOSOME | 0.50 | 1.24 | 1.90E-02 |
| GOBP_PHOTORECEPTOR_CELL_DIFFERENTIATION | 0.61 | 1.38 | 1.91E-02 |
| GOBP_EMBRYONIC_FORELIMB_MORPHOGENESIS | 0.70 | 1.44 | 1.99E-02 |
| GOBP_INTERSTRAND_CROSS_LINK_REPAIR | 0.58 | 1.34 | 2.00E-02 |
| GOBP_INTRACILIARY_TRANSPORT | 0.57 | 1.34 | 2.00E-02 |
| GOBP_POSITIVE_REGULATION_OF_CILIUM_ASSEMBLY | 0.61 | 1.38 | 2.01E-02 |
| GOBP_NEGATIVE_REGULATION_OF_T_CELL_PROLIFERATION | 0.55 | 1.31 | 2.10E-02 |
| GOBP_POSITIVE_REGULATION_OF_SIGNAL_TRANSDUCTION_BY_P53_CLASS_MEDIATOR | 0.60 | 1.37 | 2.12E-02 |
| GOBP_EYE_PHOTORECEPTOR_CELL_DEVELOPMENT | 0.69 | 1.44 | 2.19E-02 |
| GOBP_PROTEIN_LOCALIZATION_TO_CONDENSED_CHROMOSOME | 0.65 | 1.41 | 2.25E-02 |
| GOBP_DNA_DEPENDENT_DNA_REPLICATION | 0.47 | 1.18 | 2.30E-02 |
| GOBP_PROTEIN_LOCALIZATION_TO_CYTOSKELETON | 0.53 | 1.28 | 2.30E-02 |
| GOBP_TRNA_METHYLATION | 0.55 | 1.30 | 2.30E-02 |
| GOBP_SNRNA_TRANSCRIPTION | 0.64 | 1.41 | 2.34E-02 |
| GOBP_DOUBLE_STRAND_BREAK_REPAIR | 0.45 | 1.14 | 2.60E-02 |
| GOBP_NUCLEAR_CHROMOSOME_SEGREGATION | 0.45 | 1.14 | 2.60E-02 |
| GOBP_POSITIVE_REGULATION_OF_CHROMOSOME_ORGANIZATION | 0.50 | 1.23 | 2.60E-02 |
| GOBP_B_CELL_ACTIVATION | 0.46 | 1.16 | 2.70E-02 |
| GOBP_PROTEIN_K63_LINKED_DEUBIQUITINATION | 0.56 | 1.32 | 2.70E-02 |
| GOBP_KINETOCHORE_ORGANIZATION | 0.62 | 1.38 | 2.74E-02 |
| GOBP_HISTONE_H3_K9_TRIMETHYLATION | 0.66 | 1.41 | 2.76E-02 |
| GOBP_CHROMOSOME_SEGREGATION | 0.44 | 1.14 | 2.80E-02 |
| GOBP_REGULATION_OF_PROTEIN_LOCALIZATION_TO_CILIUM | 0.68 | 1.42 | 2.82E-02 |
| GOBP_HYDROGEN_PEROXIDE_CATABOLIC_PROCESS | -0.49 | -1.81 | 2.94E-02 |
| GOBP_RECOMBINATIONAL_REPAIR | 0.47 | 1.18 | 3.00E-02 |
| GOBP_NEGATIVE_REGULATION_OF_DNA_BIOSYNTHETIC_PROCESS | 0.57 | 1.31 | 3.00E-02 |
| GOBP_NEGATIVE_REGULATION_OF_DNA_RECOMBINATION | 0.57 | 1.32 | 3.00E-02 |
| GOBP_CYCLIC_NUCLEOTIDE_METABOLIC_PROCESS | 0.63 | 1.39 | 3.05E-02 |
| GOBP_MEIOTIC_CHROMOSOME_SEGREGATION | 0.53 | 1.26 | 3.10E-02 |
| GOBP_SNRNA_METABOLIC_PROCESS | 0.53 | 1.27 | 3.10E-02 |
| GOBP_NEGATIVE_REGULATION_OF_PLASMA_MEMBRANE_BOUNDED_CELL_PROJECTION_ASSEMBLY | 0.60 | 1.35 | 3.13E-02 |
| GOBP_THYMIC_T_CELL_SELECTION | 0.67 | 1.41 | 3.14E-02 |
| GOBP_NEGATIVE_REGULATION_OF_LEUKOCYTE_CELL_CELL_ADHESION | 0.49 | 1.21 | 3.20E-02 |
| GOBP_CYTOPLASMIC_PATTERN_RECOGNITION_RECEPTOR_SIGNALING_PATHWAY | 0.53 | 1.27 | 3.20E-02 |
| GOBP_NEGATIVE_REGULATION_OF_TELOMERE_MAINTENANCE_VIA_TELOMERE_LENGTHENING | 0.59 | 1.34 | 3.23E-02 |
| GOBP_SISTER_CHROMATID_COHESION | 0.52 | 1.26 | 3.30E-02 |
| GOBP_B_CELL_RECEPTOR_SIGNALING_PATHWAY | 0.52 | 1.25 | 3.50E-02 |
| GOBP_ACTIVATION_OF_PROTEIN_KINASE_B_ACTIVITY | 0.62 | 1.37 | 3.55E-02 |
| GOBP_ANTIGEN_RECEPTOR_MEDIATED_SIGNALING_PATHWAY | 0.47 | 1.17 | 3.60E-02 |
| GOBP_NEGATIVE_REGULATION_OF_DNA_REPLICATION | 0.58 | 1.32 | 3.61E-02 |
| GOBP_NEGATIVE_REGULATION_OF_FIBROBLAST_PROLIFERATION | 0.59 | 1.33 | 3.61E-02 |
| GOBP_ALPHA_BETA_T_CELL_ACTIVATION | 0.48 | 1.19 | 3.90E-02 |
| GOBP_PROTEIN_LOCALIZATION_TO_CHROMATIN | 0.56 | 1.30 | 3.90E-02 |
| GOBP_RNA_METHYLATION | 0.49 | 1.21 | 4.00E-02 |
| GOBP_DEFENSE_RESPONSE_TO_FUNGUS | 0.61 | 1.35 | 4.08E-02 |
| GOBP_PEPTIDYL_SERINE_MODIFICATION | 0.44 | 1.13 | 4.10E-02 |
| GOBP_REGULATION_OF_AMPA_RECEPTOR_ACTIVITY | -0.43 | -1.50 | 4.26E-02 |
| GOBP_KINETOCHORE_ASSEMBLY | 0.63 | 1.36 | 4.30E-02 |
| GOBP_RELAXATION_OF_MUSCLE | 0.61 | 1.35 | 4.39E-02 |
| GOBP_NEGATIVE_REGULATION_OF_DOUBLE_STRAND_BREAK_REPAIR_VIA_HOMOLOGOUS_RECOMBINATION | 0.64 | 1.37 | 4.44E-02 |
| GOBP_GOLGI_TO_ENDOSOME_TRANSPORT | 0.62 | 1.36 | 4.48E-02 |
| GOBP_NUCLEOTIDE_BINDING_DOMAIN_LEUCINE_RICH_REPEAT_CONTAINING_RECEPTOR_SIGNALING_PATHWAY | 0.61 | 1.34 | 4.49E-02 |
| GOBP_DNA_DOUBLE_STRAND_BREAK_PROCESSING | 0.65 | 1.37 | 4.50E-02 |
| GOBP_HISTONE_MRNA_METABOLIC_PROCESS | 0.58 | 1.31 | 4.52E-02 |
| GOBP_GOLGI_ORGANIZATION | 0.47 | 1.16 | 4.60E-02 |
| GOBP_TRANSLESION_SYNTHESIS | 0.59 | 1.33 | 4.75E-02 |
| GOBP_REGULATION_OF_MYOBLAST_PROLIFERATION | 0.68 | 1.39 | 4.77E-02 |
| GOBP_REGULATION_OF_CILIUM_ASSEMBLY | 0.50 | 1.21 | 4.80E-02 |
| GOBP_PROTEIN_LOCALIZATION_TO_CHROMOSOME_CENTROMERIC_REGION | 0.59 | 1.32 | 4.83E-02 |
| GOBP_FORELIMB_MORPHOGENESIS | 0.63 | 1.35 | 4.86E-02 |
| GOBP_SNRNA_TRANSCRIPTION_BY_RNA_POLYMERASE_II | 0.64 | 1.36 | 4.86E-02 |
| GOBP_REGULATION_OF_HISTONE_H3_K9_METHYLATION | 0.60 | 1.33 | 4.96E-02 |
| GOCC_CENTRIOLE | 0.61 | 1.53 | 9.99E-04 |
| GOCC_CUL3_RING_UBIQUITIN_LIGASE_COMPLEX | 0.63 | 1.47 | 1.00E-03 |
| GOCC_CILIARY_BASAL_BODY | 0.51 | 1.27 | 2.00E-03 |
| GOCC_CIS_GOLGI_NETWORK | 0.58 | 1.39 | 5.00E-03 |
| GOCC_IMMUNOGLOBULIN_COMPLEX | 0.57 | 1.36 | 8.00E-03 |
| GOCC_CILIARY_TRANSITION_ZONE | 0.56 | 1.33 | 1.10E-02 |
| GOCC_PML_BODY | 0.51 | 1.25 | 1.20E-02 |
| GOCC_EXTERNAL_SIDE_OF_PLASMA_MEMBRANE | 0.46 | 1.18 | 1.30E-02 |
| GOCC_SPINDLE_POLE | 0.48 | 1.20 | 1.50E-02 |
| GOCC_MYOSIN_II_COMPLEX | -0.61 | -1.91 | 1.72E-02 |
| GOCC_CILIARY_PLASM | 0.52 | 1.28 | 1.80E-02 |
| GOCC_METHYLOSOME | -0.46 | -1.62 | 2.13E-02 |
| GOCC_CONDENSED_CHROMOSOME | 0.46 | 1.17 | 2.20E-02 |
| GOCC_RESPIRATORY_CHAIN_COMPLEX_III | -0.51 | -1.83 | 2.38E-02 |
| GOCC_PHOTORECEPTOR_INNER_SEGMENT | 0.58 | 1.32 | 2.40E-02 |
| GOCC_NON_MOTILE_CILIUM | 0.50 | 1.22 | 3.00E-02 |
| GOCC_DENSE_CORE_GRANULE | 0.67 | 1.41 | 3.14E-02 |
| GOCC_MITOCHONDRIA_ASSOCIATED_ENDOPLASMIC_RETICULUM_MEMBRANE | 0.62 | 1.38 | 3.37E-02 |
| GOCC_CYTOPLASMIC_MICROTUBULE | 0.51 | 1.24 | 3.60E-02 |
| GOCC_CHROMOSOMAL_REGION | 0.44 | 1.13 | 3.70E-02 |
| GOCC_MKS_COMPLEX | 0.65 | 1.38 | 4.38E-02 |
| GOCC_ENDOCYTIC_VESICLE_LUMEN | -0.46 | -1.50 | 4.44E-02 |
| GOCC_SITE_OF_DOUBLE_STRAND_BREAK | 0.50 | 1.22 | 4.60E-02 |
| GOMF_UBIQUITIN_LIKE_PROTEIN_SPECIFIC_PROTEASE_ACTIVITY | 0.53 | 1.31 | 9.99E-04 |
| GOMF_RNA_BINDING_INVOLVED_IN_POSTTRANSCRIPTIONAL_GENE_SILENCING | 0.80 | 1.66 | 1.04E-03 |
| GOMF_DNA_BINDING_TRANSCRIPTION_REPRESSOR_ACTIVITY | 0.48 | 1.22 | 4.00E-03 |
| GOMF_PROTEIN_SERINE_KINASE_ACTIVITY | 0.46 | 1.17 | 5.00E-03 |
| GOMF_CYSTEINE_TYPE_PEPTIDASE_ACTIVITY | 0.49 | 1.22 | 7.99E-03 |
| GOMF_G_QUADRUPLEX_DNA_BINDING | 0.74 | 1.52 | 1.06E-02 |
| GOMF_OMEGA_PEPTIDASE_ACTIVITY | 0.51 | 1.26 | 1.30E-02 |
| GOMF_SINGLE_STRANDED_RNA_BINDING | 0.52 | 1.26 | 1.30E-02 |
| GOMF_POLY_PYRIMIDINE_TRACT_BINDING | 0.62 | 1.40 | 1.51E-02 |
| GOMF_PROTEIN_SERINE_THREONINE_KINASE_ACTIVITY | 0.44 | 1.14 | 1.60E-02 |
| GOMF_ENDODEOXYRIBONUCLEASE_ACTIVITY | 0.59 | 1.36 | 1.60E-02 |
| GOMF_PHOSPHATIDYLINOSITOL_KINASE_ACTIVITY | 0.67 | 1.47 | 1.63E-02 |
| GOMF_POLY_PURINE_TRACT_BINDING | 0.60 | 1.36 | 2.11E-02 |
| GOMF_LYS63_SPECIFIC_DEUBIQUITINASE_ACTIVITY | 0.68 | 1.42 | 2.41E-02 |
| GOMF_TRNA_METHYLTRANSFERASE_ACTIVITY | 0.57 | 1.33 | 2.60E-02 |
| GOMF_CYCLIN_BINDING | 0.57 | 1.33 | 2.70E-02 |
| GOMF_TRNA_GUANINE_METHYLTRANSFERASE_ACTIVITY | 0.67 | 1.42 | 2.81E-02 |
| GOMF_SUMO_LIGASE_ACTIVITY | 0.70 | 1.44 | 2.86E-02 |
| GOMF_DNA_DIRECTED_DNA_POLYMERASE_ACTIVITY | 0.61 | 1.36 | 3.04E-02 |
| GOMF_HELICASE_ACTIVITY | 0.47 | 1.18 | 3.10E-02 |
| GOMF_S_ACYLTRANSFERASE_ACTIVITY | 0.58 | 1.32 | 3.31E-02 |
| GOMF_SUMO_TRANSFERASE_ACTIVITY | 0.61 | 1.36 | 3.35E-02 |
| GOMF_OLIGOPEPTIDE_BINDING | -0.55 | -1.73 | 3.45E-02 |
| GOMF_GDP_DISSOCIATION_INHIBITOR_ACTIVITY | 0.64 | 1.39 | 3.47E-02 |
| GOMF_ANTIGEN_BINDING | 0.50 | 1.22 | 3.60E-02 |
| GOMF_MODIFICATION_DEPENDENT_PROTEIN_BINDING | 0.46 | 1.16 | 3.70E-02 |
| GOMF_CATALYTIC_ACTIVITY_ACTING_ON_DNA | 0.46 | 1.16 | 3.80E-02 |
| GOMF_PHOSPHATIDYLINOSITOL_3_KINASE_ACTIVITY | 0.69 | 1.40 | 4.03E-02 |
| GOMF_SOLUTE_SODIUM_SYMPORTER_ACTIVITY | 0.61 | 1.35 | 4.08E-02 |
| GOMF_MANNOSIDASE_ACTIVITY | 0.63 | 1.36 | 4.39E-02 |
| GOMF_PROTEIN_TAG | -0.46 | -1.49 | 4.44E-02 |
| GOMF_NEUTRAL_AMINO_ACID_TRANSMEMBRANE_TRANSPORTER_ACTIVITY | 0.56 | 1.29 | 4.81E-02 |
| KEGG_NON_HOMOLOGOUS_END_JOINING | 0.73 | 1.51 | 3.12E-03 |
| KEGG_PHENYLALANINE_METABOLISM | -0.55 | -1.73 | 1.82E-02 |
| KEGG_RIG_I_LIKE_RECEPTOR_SIGNALING_PATHWAY | 0.53 | 1.27 | 2.60E-02 |
| KEGG_PRIMARY_IMMUNODEFICIENCY | 0.59 | 1.32 | 4.57E-02 |
