# Supplementary figures and images for: Integrative analyses of a mitophagy-related gene signature for predicting prognosis in patients with uveal melanoma
Source: Front Genet. 2022 Dec 5;13:1050341. doi: 10.3389/fgene.2022.1050341 (PMC9760814; doi:10.3389/fgene.2022.1050341)

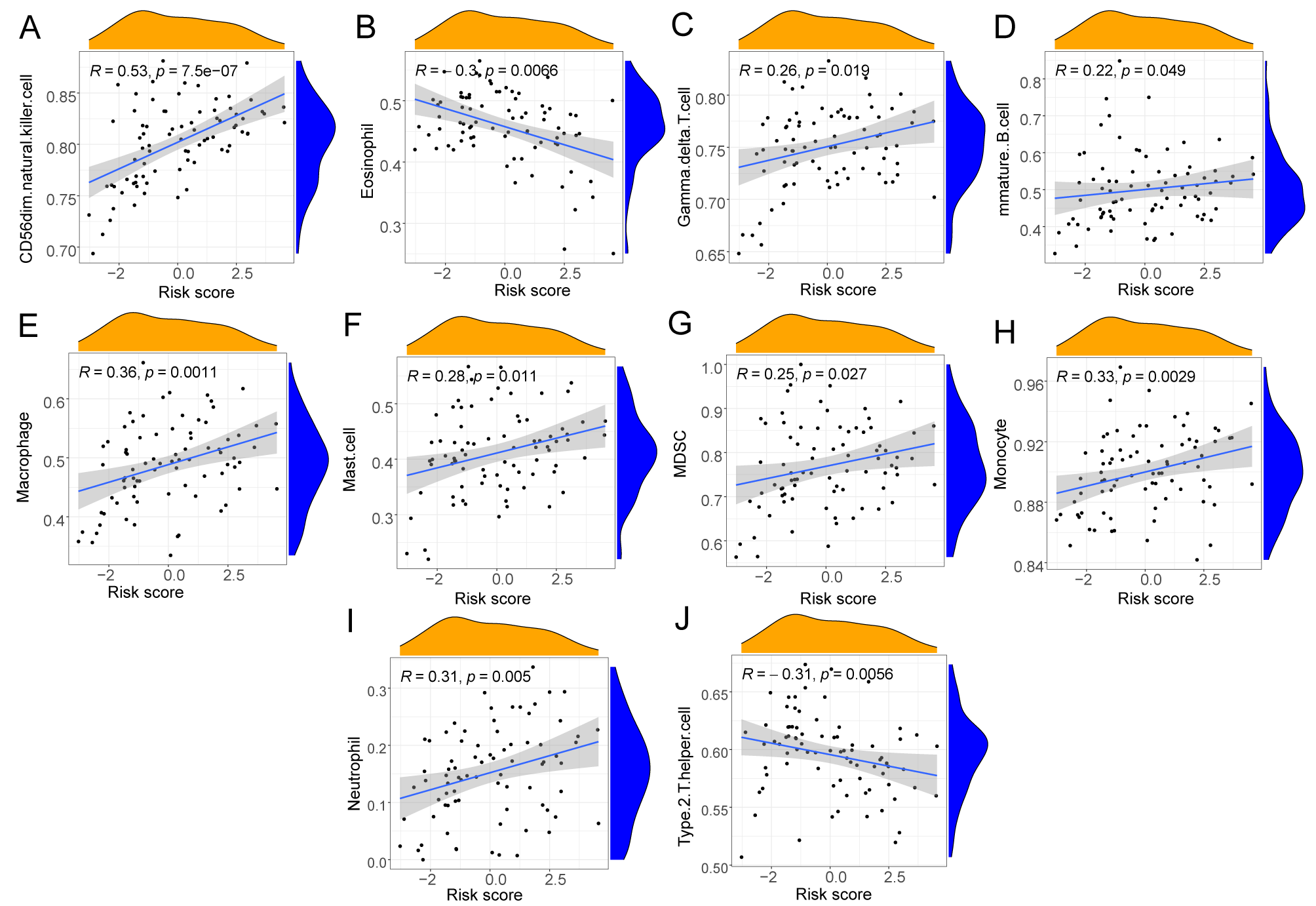

Supplement: Supplementary file 2 [file Image2.TIF]

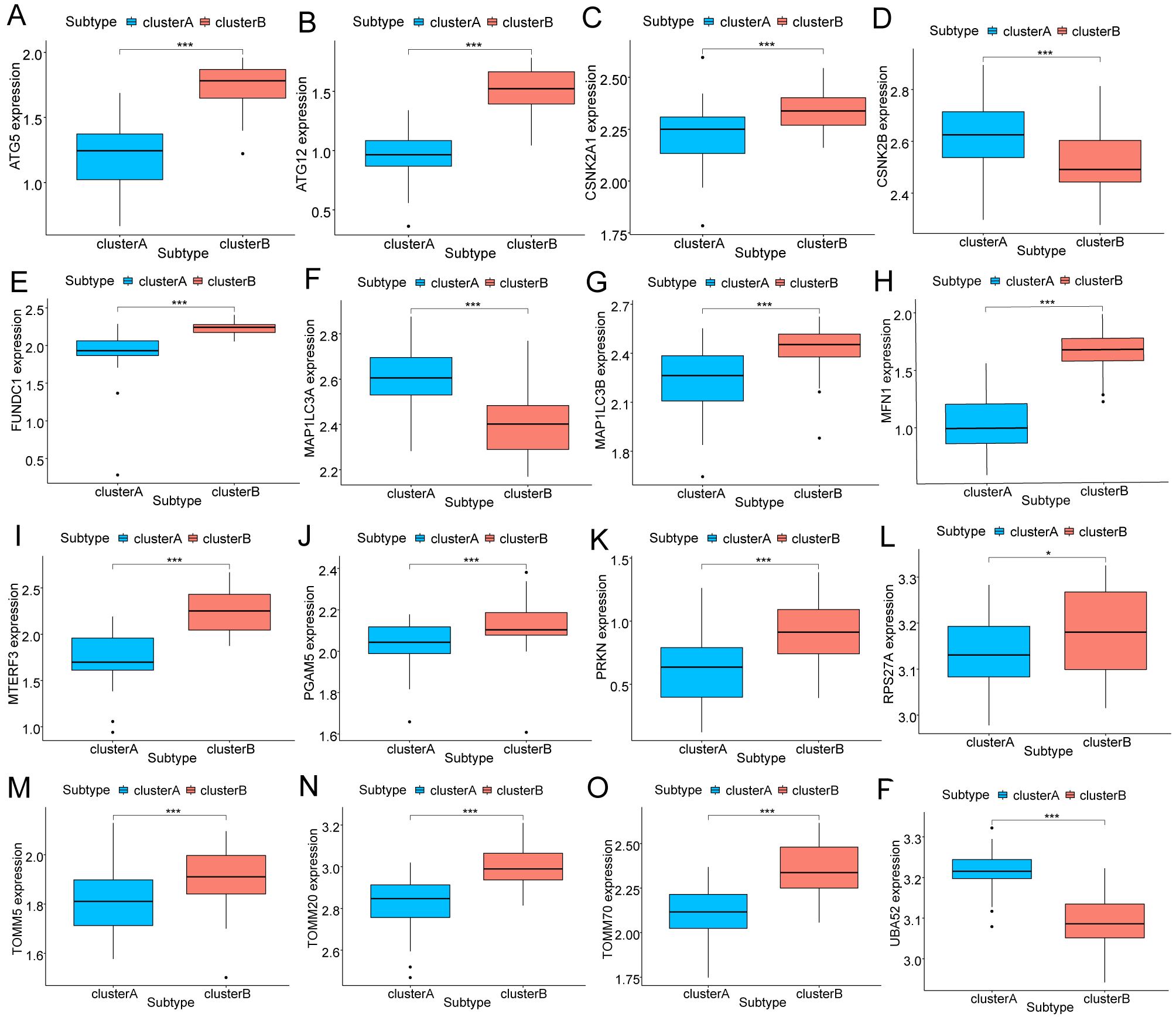

Supplement: Supplementary file 3 [file Image1.TIF]
